# Implications of Breast Cancer Chemotherapy-Induced Inflammation on the Gut, Liver, and Central Nervous System

**DOI:** 10.3390/biomedicines9020189

**Published:** 2021-02-13

**Authors:** Taurean Brown, DeLawrence Sykes, Antiño R. Allen

**Affiliations:** 1 Division of Radiation Health, University of Arkansas for Medical Sciences, Little Rock, AR 72205, USA; TBrown8@uams.edu; 2 Department of Pharmaceutical Sciences, University of Arkansas for Medical Sciences, Little Rock, AR 72205, USA; 3 Department of Neurobiology & Developmental Sciences, University of Arkansas for Medical Sciences, Little Rock, AR 72205, USA; 4 Department of Biology, Pomona College, Claremont, CA 91711, USA; delawerence.sykes@pomona.edu

**Keywords:** cyclophosphamide, doxorubicin, docetaxel, paclitaxel

## Abstract

Breast Cancer is still one of the most common cancers today; however, with advancements in diagnostic and treatment methods, the mortality and survivorship of patients continues to decrease and increase, respectively. Commonly used treatments today consist of drug combinations, such as doxorubicin and cyclophosphamide; docetaxel, doxorubicin, and cyclophosphamide; or doxorubicin, cyclophosphamide, and paclitaxel. Although these combinations are effective at destroying cancer cells, there is still much to be understood about the effects that chemotherapy can have on normal organ systems such as the nervous system, gastrointestinal tract, and the liver. Patients can experience symptoms of cognitive impairments or “chemobrain”, such as difficulty in concentrating, memory recollection, and processing speed. They may also experience gastrointestinal (GI) distress symptoms such as diarrhea and vomiting, as well as hepatotoxicity and long term liver damage. Chemotherapy treatment has also been shown to induce peripheral neuropathy resulting in numbing, pain, and tingling sensations in the extremities of patients. Interestingly, researchers have discovered that this array of symptoms that cancer patients experience are interconnected and mediated by the inflammatory response.

## 1. Introduction 

Breast cancer is one of the most common cancers in the world and one of the leading causes of mortality among women worldwide [1]. Although incidences of breast cancer are increasing in countries such as the United States, related mortality continues to decrease, and survivorship continues to increase [1]. In 1975, the breast cancer death rate for women aged 30 to 79 was 48.3 deaths per 100,000 women; however, by the year 2000, the breast cancer death rate dropped to 38.0 deaths per 100,000 women [2]. In the year 2010, the average annual percentage change for breast cancer related deaths decreased by 2.9 percent and this trend is expected to continue well on into the year 2030 [3]. These recent trends are largely due to improved prevention, detection, and treatment methods over the last several decades. However, despite these positive trends in survivorship, we are still trying to understand the implications of current treatment methods on the long-term health and quality of life of breast cancer patients. 

One of the oldest and most commonly used treatment methods for breast cancer and cancer, in general, is chemotherapy. Using chemotherapy to treat cancer has a long history that has its underpinnings in the 1940s, based on observations made previously during the First World War. Mustard gas, which had been used as a biological weapon, was observed to reduce white blood cell counts in soldiers, and consequently, scientists theorized that this agent could be used to treat cancer. As a result, experiments began to assess the ability of mustard agents to treat lymphoma bearing mice [4,5]. Upon noticeable regressions of the lymphoma in the mice, a less volatile form of mustard gas (mustine) was used to treat a human patient with non-Hodgkin’s lymphoma with some limited success [6]. In the decades following, chemotherapy garnered skepticism as methods such as surgery and radiotherapy were prioritized for treating cancer; however, with the eventual plateau of cure rates by these other methods and the continued development of chemotherapy treatments, chemotherapy soon began to develop into what we know today. 

Breast cancer was largely responsible for the resurgence of the use of chemotherapy both in conjunction with and after surgery. In the 1970s, chemotherapy was adapted for breast cancer with the first reports of the efficacy of combining cyclophosphamide, methotrexate, and fluorouracil (CMF) as an adjuvant treatment [4,5]. This discovery was important not only for the management of breast cancer but also for the practice of implementing drug combinations into cancer treatment as a supplement to surgery. Although the use of anthracycline agents (doxorubicin) to treat metastatic breast cancer was described in the 1960s, it was only in the 1990s that the combination of doxorubicin and cyclophosphamide (AC) became a standard treatment regimen in breast cancer treatment [7,8]. The rationale for introducing anthracyclines in breast cancer treatment was to reduce the duration of treatment, the number of hospital visits, nausea, and to improve the efficacy of CMF treatment [9]. Development of taxanes similarly began in the 1970s; however, the inclusion of drugs such as paclitaxel and docetaxel in combination with anthracycline treatments did not occur until the late 1990s and early 2000s [10,11,12,13,14]. The addition of taxanes in conjunction with AC treatment has been shown to significantly improve the efficacy of treatment and the overall survival among women with breast cancer compared to just AC alone [14,15].

Today, the most commonly used breast cancer treatments consist of one of two combination treatments: docetaxel (Taxotere), doxorubicin (Adriamycin), and cyclophosphamide (TAC) or doxorubicin, cyclophosphamide, and paclitaxel (Taxol) (AC-T). Despite the substantial evidence for the effectiveness of these combinations in treating breast cancer, much is still unknown about the long-term implications of these treatment methods on patient health. A prime example of this is the phenomenon of chemotherapy-induced cognitive impairment colloquially known as “chemobrain.” Chemobrain is defined by the experience of cognitive deficits such as impaired processing speed, memory retention, and concentration after or during chemotherapy treatment. Currently, the mechanisms behind the neurobiological damage that induces chemobrain are still not fully understood. Recent research has provided evidence to suggest that the inflammation response plays an essential role in chemotherapy-induced behavioral comorbidities as well as a host of other neurological disorders [16]. In this review, we will discuss the relationship between breast cancer chemotherapy-induced inflammation and the central and peripheral nervous systems with a particular focus on the effect of peripheral-organ inflammation on neurological outcomes. Additionally, we will discuss a few current potential anti-inflammatory therapeutic options that may help attenuate neurological deficits inflicted by breast cancer chemotherapy. 

## 2. Chemobrain and Inflammation: What Is the Connection? 

Chemobrain is a condition in cancer patients described as cognitive impairment or decline following chemotherapy treatment. Initial concerns over the cognitive deficits associated with chemotherapeutics began as early as the late 1950s and accelerated in the 1980s as more evidence for this condition was identified [17,18]. Over the last 40 years, chemobrain has remained a concern in cancer patients as evidenced by the continued research on the phenomenon [19]. It is estimated that between 13% and 78% of breast cancer patients report experiencing measurable cognitive impairment both during and after chemotherapy treatment [20,21,22]. Typically, most breast cancer patients report a wide range of cognitive impairment, which includes deficits in memory retention, executive function, reaction time, and processing speed. Longitudinal studies have also shown that chemobrain symptoms can persist as long as 2 to 20 years after chemotherapy treatment [23,24,25,26,27]. Similar behavioral deficits have been observed in animal models. For example, several studies using the Y-maze spontaneous alternation task have observed deficits in short-term memory in rodent models after administration of both cyclophosphamide and doxorubicin [28,29]. Other studies have assessed short-term memory using the novel object recognition test, which tests a rodent’s natural curiosity to investigate novel stimuli, and found a lowered preference score for the novel object in rodents administered a cocktail of CMF [30]. Similarly, impairments in long-term spatial memory have been observed using the Morris Water Maze in rodents that were administered either CMF or AC-T [31,32]. 

Despite this knowledge, there is currently a lack of understanding of the underlying mechanisms that induce the chemobrain phenotype. The secondary damage that chemotherapeutics can enact on the body and its various organ systems has been well established in the literature. Though they are effective at killing cancer, many current treatments lack the specificity to only attack cancer cells and consequently inflict damage on normal healthy tissue. For example, doxorubicin, a commonly used drug in breast cancer treatment, has long been implicated in inducing cumulative and dose-dependent cardiotoxicity resulting in severe cardiomyopathy or congestive heart failure through mechanisms such as apoptosis, oxidative stress, and mitochondrial damage [33,34,35].

Unfortunately, the central nervous system (CNS) is not entirely exempt from these untargeted side effects of chemotherapeutics. One innate form of defense the brain has against direct non-targeted tissue damage from chemotherapeutic drugs is the blood–brain barrier (BBB), a microvascular semi-permeable filtration system of the CNS that prevents the passage of pathogens and toxins from the bloodstream into the brain [36]. This normally beneficial barrier can also present an obstacle to the delivery of drugs to the central nervous system, such as in the treatment of brain cancers. To bypass this limitation, several different methods have been developed to increase the permeability of drugs through the BBB [37]. However, in cases where we do not want to directly treat or affect the CNS, such as in breast cancer treatment, we still observe damage or impairments in CNS functions such as chemobrain. Generally, most commonly used breast cancer chemotherapeutics such as doxorubicin and taxanes do not readily cross the BBB with the exception of cyclophosphamide and fluorouracil [38,39,40,41]. Since most of these drugs cannot damage the CNS directly, the sustaining damage they inflict comes indirectly through various mechanisms, most notably inflammation. 

**Figure 1:**
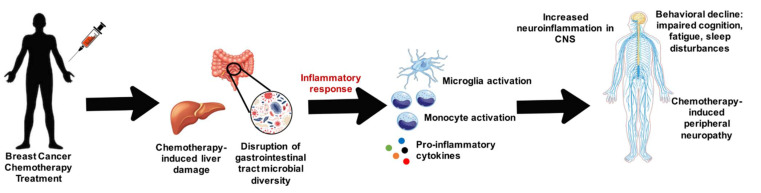
Overview of how breast cancer chemotherapy can damage the liver and disrupt microbial diversity within the gastrointestinal tract and cause inflammatory mediated damage on the nervous system.

Inflammation is the body’s normal immune response to external damage, pathogens, or chemical or radioactive irritants. The inflammatory response is mediated predominantly by cytokines, small protein signaling molecules responsible for regulating inflammation in response to infection, injury, or wound healing. Cytokine dysregulation and subsequent sustained inflammation is an important factor in many disease states, such as cardiovascular disease, pulmonary disease, cancer, and neurological disorders [42]. Chemotherapy has been implicated in inducing inflammation by disrupting normal cytokine regulation in both human and rodent models as well as inducing monocytic migration to areas of inflammation both within the body and the CNS [43,44]. Migrating monocytes can cluster and produce pro-inflammatory cytokines resulting in dysregulation of normal cytokine production. Cytokine dysregulation is a detrimental feature of cancer treatment, as the chemotherapy can result in damage to all tissues, inflicting sustained damage to normal tissue both directly and indirectly. Recently, researchers have begun to investigate this relationship between peripheral inflammation in various organ systems and the brain to explain the mechanisms behind conditions such as chemobrain. In the next few sections, we will discuss how chemotherapy can induce inflammation in peripheral organs such as the gut and liver and cause neurological comorbidities (Figure 1). 

## 3. Gut-Brain Axis and Inflammation 

The human gastrointestinal (GI) tract contains a complex ecosystem of microbes estimated to number over 1014 [45]. The microbes that inhabit the GI tract collectively encode 100 times more unique genes than the human genome. These microbes expand on the metabolic capabilities found within the human genome and significantly influence neurology, immunity, endocrinology, disease states, and clinical outcomes [46]. The ability of the CNS to affect the GI tract has long been characterized; however, the ability of the microbiota within the GI tract to impact CNS functioning is a relatively new concept. Links between the microbiota of the GI tract and the brain have been observed in various neurological disorders, such as Parkinson’s disease, schizophrenia, autism, depression, anxiety, and Alzheimer’s disease [47,48,49,50]. 

The connection between the GI tract and the nervous system are currently explained fundamentally by disruptions in the microbial diversity within the gut. Different microbes existing within the gut produce byproducts that are essential for normal bodily homeostasis as well as regulation of metabolic and immune processes. In cancer patients, chemotherapy often results in the reduction of important microbes within the gut such as the butyrate-producing bacteria Faecalibacterium and Roseburia [51]. Butyrate is a microbial byproduct that has anti-inflammatory properties. The effects of chemotherapy have been examined in numerous animal models. Although rodents have a different microbial composition, a similar pathophysiology can be characterized in mice and humans after chemotherapy, including, crypt ablation, villus blunting, epithelial atrophy of the small and large intestine with accompanied mucosal damage and mucosal degradation [52,53]. One study found that chemotherapeutic drugs increased β-glucuronidase-producing bacteria in a rodent model, causing the reactivation of chemotherapeutics in the GI tract and contributing to intestinal toxicity, mucositis, and diarrhea [54]. Changes in GI microbial composition have also been observed in various neurological conditions. For instance, in a mouse model of Alzheimer’s disease, animals had a decrease in Allobaculum and Akkermansia and an increase in Rikenellaceae relative to the wild type control animals [55]. Similar findings were observed in a clinical study that reported notable changes in the microbial diversity in the guts of Alzheimer patients, which was marked by a decrease in Firmicutes and an increase in Bacteroidetes [56]. Chemotherapy has frequently been linked to gastrointestinal complications due to a variety of symptoms in cancer patients such as diarrhea, constipation, and vomiting [57,58,59]. The severity of these types of symptoms can result in dosage adjustments, delays in treatments, or discontinuation of treatments, causing poor clinical outcomes. 

In addition to the disruption of gut microflora, these chemotherapeutic agents can also produce intestinal inflammation. Gastrointestinal inflammation has been implicated extensively as the link between the gut and CNS. One proposed mechanism of action is that decreases in microbial diversity and irritation of the intestinal lining subsequently results in the increased permeability of the intestines. This can result in infiltration of peripheral immune cells into the intestine, causing activation of immune cells and the release of pro-inflammatory cytokines that induce peripheral inflammation as well as neuroinflammation in the brain, resulting in behavioral impairments [60]. Neuroinflammation is one of the primary mechanisms thought to underlie long-term cognitive dysfunction in aging and neurological disorders such as chemobrain [61]. Peripheral cytokines released from the gut and nearby tissue as a result of chemotherapy are thought to travel through the bloodstream, bypassing the BBB, and directly inducing inflammation in brain tissue. Some research even suggests that this prolonged cytokine expression could damage the integrity of the BBB allowing for chemotherapeutic agents to more readily pass and damage brain tissue directly [62]. The gut microbiota have a dual role being both helpful and harmful as a consequence of the dysbiosis caused by chemotherapeutic treatment. Certain clusters can contribute to pathophysiology as a secondary effect of treatment. Chemotherapy induced mucosal inflammation of the gastrointestinal tract, termed mucositis, adversely impacts some intestinal microbes. For example, conventional mice show increased inflammation and higher intestinal epithelial permeability compared to germ free mice post mucositis induction [63]. The increased inflammation and permeability results from an increased number of lesions in the epithelium of conventional mice which ultimately increased susceptibility to the harmful effects of some gut microbes that opt to be facultatively opportunistic. These results provide evidence for the key role that gut microbes play in the development and progression of mucositis.

Gut microbiota complexity is also suspected to either directly or indirectly influence microglia, the immune cells within the CNS. Germ-free mice exhibited impairments in microglial maturation and function that were restored when microbiota or short fatty chain acids were introduced into the mice [64]. In addition to increased cytokine expression, increased microglial activation in brain regions responsible for mood regulation and cognition have been observed in behavioral disorders such as major depression disorder [65,66]. Peripheral cytokine expression induced by chemotherapy is also suspected to induce localized neuroinflammation by stimulating/activating other neuronal cells, such as astrocytes, oligodendrocytes, and neurons resulting in localized cytokine/chemokine release and consequent cognitive impairment [67]. 

## 4. Liver-Brain Inflammation Axis 

The connection between the liver and the brain is another emerging area of research as is the liver’s contribution to chemobrain. The liver is unique in that it services as a barrier between the gut and the body; additionally, peripheral organ centered inflammation changes neural transmission of the CNS thereby altering behavior [68]. Several mechanisms have been elucidated for crosstalk between the liver and the brain, underscoring the role the liver plays in facilitating communication between the brain and the periphery. Such as the GI tract, the connection between the liver and brain is suspected to be mediated by the immune system due to acute liver damage. Chemotherapy-induced hepatotoxicity has long been a concern in cancer treatment. This is because many chemotherapeutic drugs require optimal liver functioning in order to be metabolized, which can consequently induce liver damage. Several common breast cancer drugs, such as methotrexate, doxorubicin, and cyclophosphamide have been cited with various levels of hepatotoxicity [69]. Cyclophosphamide and doxorubicin have also been implicated in causing drug-induced liver injury conditions such as sinusoidal obstruction syndrome [70,71]. Even taxanes such as docetaxel and paclitaxel as well as 5 F-U have been found to induce liver injury through the accumulation of fat globules in hepatocytes [71]. Most chemotherapeutic drugs are lipophilic and are readily taken up by the liver, which can result in irreversible hepatocellular damage through the recruitment of inflammatory cells [69]. Another unique feature of the liver is reflected in the heterogeneous nature of its cellular composition. Roughly 80% of cells in the liver are hepatocytes with the remaining cells consisting of non-parenchymal cells such as intracellular hepatocytes, stellate cells, and Kupffer cells [68]. The liver contains the largest population of Kupffer cells (a type of macrophage) in the body. Liver inflammation is usually characterized by the activation of Kupffer cells and the production of pro-inflammatory cytokines, such as NF-α, IL-1β, and IL-6 [72,73]. The liver’s peripheral connection to the brain in the context of inflammation has been described in four different pathways. First, the neural pathway describes the connection between the CNS and the liver via the vagus nerve. The liver is innervated by vagal afferents that can respond to immune mediators such as proinflammatory cytokines [74,75]. Vagal nerve afferents express cytokine receptors in addition to having macrophages within its fibers that can also respond to cytokines, which could directly induce inflammation in the CNS [76]. One study found that induced peripheral inflammation in human subjects resulted in increased activity within an area of the vagus during a high performance word task, which was correlated with increased fatigue [77]. However, recent studies suggest a decreased importance of this pathway in prolonged peripheral inflammation as liver transplants (requires denervation of the liver) in Hepatitis C patients have not been shown to improve long term behavioral outcomes [78]. Second, circulating cytokines released by the liver can also directly interact with receptors on cerebral endothelial cells. TNF-alpha and IL-beta mediated signaling in the liver can induce activation of NF-κB signaling resulting in signal cascade events that can activate immune cells such as microglia within the brain parenchyma inducing inflammation [68,74]. Third, circulating cytokines from the liver can also induce immune responses via circumventricular organs and the choroid plexus, regions of the brain that lack a BBB. Fourth, circulating cytokines from the liver can also trigger monocyte transmigration into the brain in response to the activation of microglia. Once activated microglia can produce the monocyte chemoattractant protein 1(MCP-1), triggering the recruitment of monocytes into the brain resulting in an inflammation cascade event [74]. Whatever the mechanism of action it is evident that chemotherapy induced liver injury is a serious issue that can directly impact other organ systems such as the CNS. Liver induced brain inflammation has also been reported to affect cognitive outcomes and cause many different behavioral comorbidities, such as fatigue, difficulty concentrating, sleep disturbances, or memory impairment [68,79,80,81]. 

## 5. Chemotherapy-Induced Peripheral Neuropathy

In addition to the aforementioned effects on the CNS, chemotherapeutic agents can also affect the sensory, motor, and autonomic functions of the peripheral nervous system (PNS). Chemotherapy-induced peripheral neuropathy (CIPN) is characterized by damage to nerves that control movement and sensory processing for extremities such as the arms, legs, and feet. CIPN usually has a range of symptoms including numbness, tingling, altered touch sensation, spontaneous painful sensations, impaired balance or movement, constipation, and impaired sexual or urinary function [82,83]. CIPN is becoming increasingly more relevant in clinical settings with reported incidences of 68.1% when measured in the first month after chemotherapy, 60.0% at 3 months, and 30.0% at and after 6 months [84,85]. Some risk factors of CIPN in cancer patients can include genetic predisposition, history of smoking, and the overall sum of chemotherapeutics received [84,85]. Several types of chemotherapeutic agents are known to induce CIPN, most notably, taxanes (paclitaxel and docetaxel), which are frequently used within breast cancer treatment [86]. Although docetaxel and paclitaxel are unable to cross the BBB, CIPN is a dose-limiting adverse side effect of treatment [86]. There are several suggested mechanisms believed to contribute to this condition; however, one prime candidate is neuronal damage by way of immune-mediated processes. Chemotherapeutics such as taxanes can induce peripheral inflammation by inducing the release of pro-inflammatory cytokines within tissues affected by the drug. Peripheral cytokine release can then activate immune-associated cells within the CNS (macrophages, monocytes, astrocytes, and microglia) causing neuroinflammation [87,88]. Once activated immune-associated cells of the CNS can cluster and increase levels of pro-inflammatory cytokines, which can result in nociceptor sensitization and hyperexcitability of peripheral neurons [89]. Evidence of this mechanism of action is further supported by a study that showed that CIPN can be prevented in paclitaxel-treated rodents by treatment with an inhibitor of macrophages, monocytes, and microglia [90]. 

This inflammatory cascade induced by taxanes may also play a role in axon degeneration, which may contribute to the CIPN phenotype [89]. One study found that a decrease in the level of the chemokine MCP and subsequent decreased activation of its receptor C Chemokine Receptor 2 (CCR2) decreases nerve degeneration as well as CIPN-such as behaviors in rodents [91]. 

## 6. Therapeutic Strategies for Chemotherapy-Induced Inflammation

The conditions caused by chemotherapy-induced inflammation and damage to the CNS and PNS are all likely multifactorial and involve several of the mechanisms outlined above as well as others not explicitly outlined in this review such as oxidative stress damage. In terms of therapeutic strategies specific to combating chemotherapy-induced inflammation, there are a few options; however, the efficacy of all of these treatments have yet to fully be determined. One strategy that researchers have implemented in order to treat chemotherapy-induced cognitive impairment is to directly target the CNS by manipulating mechanisms involved in neuroinflammation as well as restoring cognitive performance via cognitive strengthening exercises. Ginkgo biloba is one compound that has been used to treat cognitive impairments observed after breast cancer specifically [92]. Ginkgo biloba is a compound isolated from the leaves of the ginkgo tree and has been widely used over the counter for its mental health benefits as well as neuroprotective properties [93]. Ginkgo biloba neuroprotective properties stem from its manipulation of pathways such as Nrf2/HO2 and CRMP2 [94]. Both of these pathways have been implicated in manipulating inflammatory processes such as the recruitment of immune cells to sites of inflammation within the body and CNS [95,96]. Chemotherapy has also been implicated in reducing neurogenesis within the brain via oxidative or inflammatory processes, which has been linked to cognitive impairment due to decreasing in synaptic plasticity. Interestingly, ginkgo biloba has also been shown to induce neurogenesis via the CRMP2 thus this compound may be beneficial if administered simultaneously with chemotherapy treatment [94]. Non-medical approaches such as cognitive therapy for cancer patients have also become increasingly more commonplace to help patients cope with cognitive decline after treatment. This can include behavior training strategies in memory retention, attention span, self-awareness, relaxation, meditation, and computer simulated activities [97,98,99,100,101,102].

For inflammation induced within the GI tract, one method of treatment is the implementation of prebiotics, probiotics, and postbiotics supplementation. Prebiotics are nondigestible ingredients that support the growth of beneficial bacteria in the GI tract and probiotics are supplements that contain beneficial GI bacteria. Pairing probiotics with cancer treatment has been shown to ease GI issues, intestinal inflammation, and intestinal permeability while increasing the microbial diversity in cancer patients [103]. Probiotics also have been shown to improve behavioral outcomes in clinical trials of patients with depression, anxiety, and Alzheimer’s disease [104,105,106,107]. There is less research on prebiotic supplementation; however, some studies have shown evidence of improved sleep behaviors and reduction of mood comorbidities in rodents and humans respectively [108,109]. Recently, the potential of metabolite-based therapeutic strategies or “postbiotics” as a method to treat microbial disruptions within the GI tract has been investigated. It is theorized that the intestinal microbiota of the gut impact host physiology through the secretion of small metabolites that modulate intricate cellular functions of the host organism [110,111,112]. The approach of this therapy is to not specifically target microbial composition in the gut but rather administer or inhibit metabolites in order to counteract the negative side effects of microbiome disruptions [113]. In addition, some studies have implicated the importance of dietary choices and exercise in improving microbiome health and neuronal health [114,115]. For example, alpha linoleic acid (ALA) an omega-3 polyunsaturated acid isolated from plant sources such as walnuts and soybean oil has been well documented in its role in brain development, anti-inflammatory, and antioxidative activities particularly in Alzheimer’s models [116,117]. ALA diet supplementation has been noted to improve cognitive performance in Alzheimer’s models through the inhibition of pro-inflammatory cytokines as well as decreasing oxidative stress levels [118,119]. Approaching correcting disruptions in microbial communities in the GI tract from these different perspectives could potentially reduce GI tract inflammation induced in chemotherapy treatment and subsequently improve behavioral outcomes of cancer patients. 

Anti-inflammatory therapies for liver inflammation are limited; however, there has been some evidence of promise in a few drug treatments. Some studies have investigated the potential of inhibiting the recruitment of monocyte-derived, inflammatory macrophages into the liver with drugs such as cenicriviroc [120,121,122]. Chrysin has been found in rats to have a protective effect on cyclophosphamide-induced hepatotoxicity, inflammation, and apoptosis [123]. Garlic extract has been shown to have protective effects against hematological alterations, immunosuppression, hepatic oxidative stress, and renal damage in part due to decreasing cytokine levels in serum of rats [124]. Similarly, geraniol has been found to protect against cyclophosphamide-induced hepatotoxicity in rats through mediation of MAPK and PPAR-y signaling pathways by way of reducing inflammation markers [125]. Ganglion is another potential therapeutic option for cyclophosphamide-induced liver hepatotoxicity by activating Nrf2 signaling and consequently attenuating oxidative damage, inflammation, and apoptosis in rats [126]. Ganoderic acid has also been found to attenuate hepatotoxicity by reducing cytokine levels in both serum and livers within mice [127]. 

As for CIPN there are a few potential therapeutic options. As mentioned earlier, researchers suspect monocytic migration and subsequent inflammation induced by these cells as key contributors of CIPN [44]. As such, researchers have begun to investigate the potential of targeting the receptors of these monocytic cells as well as their chemokine stimulants in order to treat CIPN. However, manipulation of these mechanisms for the purpose of CIPN treatment still remains in the preclinical stage. There is some promise though in other related pathological conditions. Monoclonal antibodies have been used to target colony stimulating factor 1, which is a molecule that regulates the differentiation of macrophages and has been used to treat solid tumors with some success in clinical trials [128]. Another humanized monoclonal antibody against CX3CL1 has been used to clinically treat rheumatoid arthritis and Crohn’s disease [129]. Thus, manipulation of monocyte migration processes may be a successful avenue for CIPN treatment. Some other candidate drugs for treatment of CIPN are metformin and minocycline [130,131,132]. Metformin although widely used as an anti-diabetic drug has been shown to reduce CIPN-such as symptoms (mechanical allodynia) in rodents treated with paclitaxel [133,134]. The anti-inflammatory effect of metformin works by decreasing pro-inflammatory cytokines and suppressing macrophage activity [135]. Minocycline functions similarly by inhibiting the activation of monocytes and decreases the release of pro-inflammatory cytokines [136,137]. One pilot study conducted on breast cancer patients found that administration of the drug did not improve general paclitaxel-induced sensory neuropathy symptoms (numbness, tingling, burning pain), but did decrease the average pain score and fatigue compared to the placebo group [138]. The use of medicinal herbs as a therapeutic approach to CIPN is also a growing area of research. Rosmarinic acid, a compound isolated from the plant rosemary, has also been shown to have anti-inflammatory properties and has been used for centuries to treat inflammatory conditions such as rheumatoid arthritis [139]. Several studies have found rosmarinic acid to be quite effective at attenuating neuropathic pain within rodents via the downregulation of pro-inflammatory markers [140,141,142]. Cannabinoids are a group of compounds isolated from the Cannabis sativa plant that mediate their effects through cannabinoid receptors and are a novel therapeutic target for inflammation. Δ9-tetrahydrocannabinol (THC) and cannabidiol (CBD) are the two active cannabinoid compounds found within the plant. Cannabinoid receptors such as CB1 and CB2 are predominantly expressed in the brain and on immune cells indicating they may play a useful role in regulating inflammation [143]. There have been a few clinical studies that investigated the effectiveness of THC/CBD sprays as a treatment method for neuropathic pain within cancer patients and have observed measurable reductions in pain of patients [144,145,146]. Although the clinical efficacy of these treatments is still to be determined they are all certainly great candidates as therapeutic strategies, neuroprotectants, and anti-inflammatory agents for chemotherapy-induced inflammatory organ damage. 

## 7. Conclusions

Breast cancer is still one of the most common cancers and leading cause of mortality due to cancer in women; however, with scientific advancements in treatment and diagnostic methods, the life expectancy of breast cancer patients has improved. Despite improvements in treatment methods, such as chemotherapy, the quality of life of breast cancer patients is still a relevant topic of study as we are still discovering that some common treatments produce long-term side effects on the nervous system such as chemobrain and CIPN. More research is needed to truly understand the complex relationship mediated by the immune system between organ systems such as the GI tract, liver, and the nervous system. Continuing to delineate this complex relationship between these organ systems and the CNS will allow for the discovery of novel therapeutic approaches that will help improve the quality of life of breast cancer patient’s post-treatment. 

## Data Availability

Not applicable.
